# Determining putative vectors of the Bogia Coconut Syndrome phytoplasma using loop-mediated isothermal amplification of single-insect feeding media

**DOI:** 10.1038/srep35801

**Published:** 2016-10-27

**Authors:** Hengyu Lu, Bree A. L. Wilson, Gavin J. Ash, Sharon B. Woruba, Murray J. Fletcher, Minsheng You, Guang Yang, Geoff M. Gurr

**Affiliations:** 1 Institute of Applied Ecology, Fujian Agriculture and Forestry University, Fuzhou, Fujian 350002, China; 2 Graham Centre, Charles Sturt University, Wagga Wagga, New South Wales 2650, Australia; 3 Centre for Crop Health, University of Southern Queensland, Toowoomba, Queensland, 4350, Australia; 4 Cocoa and Coconut Research Institute, Madang, Madang Province, Papua New Guinea; 5 Graham Centre, Charles Sturt University, Orange, New South Wales 2800, Australia

## Abstract

Phytoplasmas are insect vectored mollicutes responsible for disease in many economically important crops. Determining which insect species are vectors of a given phytoplasma is important for managing disease but is methodologically challenging because disease-free plants need to be exposed to large numbers of insects, often over many months. A relatively new method to detect likely transmission involves molecular testing for phytoplasma DNA in sucrose solution that insects have fed upon. In this study we combined this feeding medium method with a loop-mediated isothermal amplification (LAMP) assay to study 627 insect specimens of 11 Hemiptera taxa sampled from sites in Papua New Guinea affected by Bogia coconut syndrome (BCS). The LAMP assay detected phytoplasma DNA from the feeding solution and head tissue of insects from six taxa belonging to four families: Derbidae, Lophopidae, Flatidae and Ricaniidae. Two other taxa yielded positives only from the heads and the remainder tested negative. These results demonstrate the utility of combining single-insect feeding medium tests with LAMP assays to identify putative vectors that can be the subject of transmission tests and to better understand phytoplasma pathosystems.

Bogia coconut syndrome (BCS) is a serious plant disease of palm species in Madang Province in Papua New Guinea (PNG). It is suspected to be associated with a phytoplasma related to, but distinct from, the coconut lethal yellowing (LY) group (16SrIV)^1^ though causality has not been demonstrated. Lethal yellowing (LY) is a devastating phytoplasma-associated disease of coconut and other palm species, At least 40 species of palms are affected globally^2^,^3^,^4^. Coconut palms (*Cocos nucifera*) showing a progression of LY-like symptoms starting with premature nut fall, then leaflet yellowing and necrosis, frond collapse and finally palm death were first identified in 2008^5^. Since this time, BCS has been causing increasingly severe losses to coconut palms in Madang Province, killing many hundreds of palms. The area affected by BCS, has expanded in recent years though it is still confined within Madang Province. Coconut is an integral part of life in the tropics and is cultivated for food and building materials as well as a providing a vital cash crop^6^,^7^. The loss of coconut plantations would result in significant economic and social upheaval in PNG^8^ as it has been shown in other parts of the world where LY has occurred^7^,^9^.

Phytoplasmas are associated with many diseases of various plant species^10^,^11^,^12^. Phytoplasmas are prokaryote plant pathogens of the class Mollicutes, discovered in 1967 and named mycoplasma-like organisms (MLOs)^13^,^14^. This relatively late discovery, and ongoing challenges in their study, stems from the fact that they cannot be cultured *in vitro* in cell-free media^2^,^6^,^15^,^16^. These phloem-limited plant pathogens are chiefly transmitted by phloem-feeding hemipteran insects^17^,^18^. Leafhopper and planthopper species are the primary phytoplasma vectors^18^,^19^,^20^,^21^,^22^,^23^,^24^, but psyllids have been reported as vectors of some phytoplasma-caused European diseases of fruit trees^25^,^26^,^27^,^28^. Some phytoplasmas can also be spread via the parasitic weed, dodder (*Cuscuta* spp.)^29^, vegetative propagation or grafting.

The most definitive method for determining the vector species within a given pathosystem is a transmission test in which infective individuals of the species are confined on initially phytoplasma-free host plants^20^,^22^,^26^,^27^,^30^. This approach for coconut has several logistical constraints^24^,^31^. Transmission tests ideally use insects from a colony maintained on host plants known (from molecular assays and symptomology) to be infected with the relevant phytoplasma. A challenge with this method is that insects need to be introduced in large numbers and over a long period of time, extending well over a year in cases where perennial plants such as coconut are used^18^. Reflecting this challenge, some studies use field-captured insects, working on the assumption that some will be infective, some will carry the phytoplasma in question as opposed to other phytoplasmas, and that this complication can be resolved later by molecular characterization. A further challenge with transmission testing is that the plants need to be caged to avoid other insects feeding upon them. When testing large plants such as coconut this demands the use of large (expensive) cages so that growth of the palm over an extended period is possible and that foliage is not in contact with the mesh walls through which wild vectors could feed. As a result of these factors, transmission tests are often not conducted at all, or with insufficient rigour, to definitively identify the vectors in most phytoplasma pathosystems involving large plant species. In contrast, it is easy to detect the presence of phytoplasmas in the body of an insect, especially with modern molecular assays. Recently, molecular biology techniques, such as loop-mediated isothermal amplification (LAMP), nucleic acid hybridization and polymerase chain reaction (PCR), have been developed for detection of non-culturable plant pathogens and identification of potential phytoplasma vector species^10^,^23^,^24^,^32^. Such detection of phytoplasma DNA in an insect, however, is not proof of vector status. Phytoplasma transmission by insect vectors is an active process with several pivotal events. After phytoplasma acquisition by insect feeding, escape from the gut to the hemocoel and subsequent penetration into, and reproduction within, the salivary glands are prerequisite to transmission^10^,^20^,^22^,^33^,^34^,^35^. The latent period is the time between when the phytoplasma is acquired by the insect and the development of an infectious titre in the salivary glands^36^. Phytoplasma DNA may be detectable in the gut of the insect or after feeding on infected plants without it being capable of transmitting the pathogen^10^,^37^,^38^. Hence, the detection of phytoplasma DNA in insects is a step toward the identification of a vector but is not evidence of its competence to transmit phytoplasmas.

An alternative, less logistically complex method for determining likely vectors is based on assaying for phytoplasma DNA in a sucrose solution upon which insects have fed^10^,^39^. In this approach, live, field-captured insects are held individually in ventilated Eppendorf tubes in which a small volume of sucrose solution is contained in the cap cavity, separated from the insect by a Parafilm barrier. Detection of phytoplasma DNA in the solution shows that the insect introduces phytoplasmas during feeding activity. Unlike transmission tests, this approach does not provide definitive proof of vector competency because neither feeding on the host plant nor acquisition of the pathogen by the plant are demonstrated. However, given the foregoing challenges associated with conducting transmission tests with large hosts such as palms, and the fact that this has precluded successful transmission tests with these in all but a few cases^3^,^40^, the feeding medium approach offers advantages. This method reduces the time for obtaining results compared with exposing insects to plants and waiting for symptoms to develop, avoids the costs of constructing large cages to enclose palms, and allows potential vector competency to be determined for individual insects. The likely importance within a given pathosystem of different vector species can be inferred based on the relative incidence of the species in the field and the proportion of positive results from assays. This method tests a large number of individuals compared with one test per plant for transmission tests so is a valuable complement to transmission tests in which putative vectors identified using the feeding medium approach can be the focus, avoiding the need to test other Hemiptera species that may be associated with diseased plants but not competent vectors.

Given the necessity of testing large numbers of samples the present study coupled – for the first time to our knowledge – the use of LAMP with the sucrose feeding medium method. Using this tandem approach we tested 627 individual insects from three BCS-affected sites and found that 6 of 11 taxa transmitted a phytoplasma on feeding and had detectable phytoplasma DNA in their head tissue, whilst two additional taxa gave positives only for head tissue. Only one phytoplasma was detected and BLAST comparisons showed it matched (100%) with selected members of the 16SrIV group. Using data on the proportion of individuals that gave a positive LAMP result from the feeding solution and field incidence of each taxon we ranked the likely importance of each insect species as a vector in this pathosystem.

## Results

### Phytoplasma detection in insect heads and single-insect feeding media using LAMP

Reflecting the poor level of morphological and molecular taxonomic information available for insects in most tropical areas, many of the taxa sampled could not be identified to named species though 11 morphologically distinct adult morphospecies of Hemiptera were evident. In addition, a morphologically distinct hemipteran nymph was relatively common in samples and this was identified as *Lophops saccharicida* (Lophopidae), one of the adult species that was also common. The Hemiptera sampled from sites were dominated by planthoppers (families Derbidae, Lophopidae, Flatidae and Ricaniidae) whilst leafhoppers (Cicadellidae) were scarce in samples (Table 1). Most taxa were present at all three of the BCS affected sites though *L. saccharicida,* the unidentified Derbidae species, and the unidentified Ricaniidae species were rare or absent on sites other than Mobdu, whilst the unidentified Flatidae species 1 was present only at Bogia.

Each of the planthopper families contained at least one lower level taxon for which molecular data suggested vector status (Table 2). In contrast, all leafhoppers, represented by two lower level taxa, tested negative for phytoplasma DNA.

**Figure 1:**
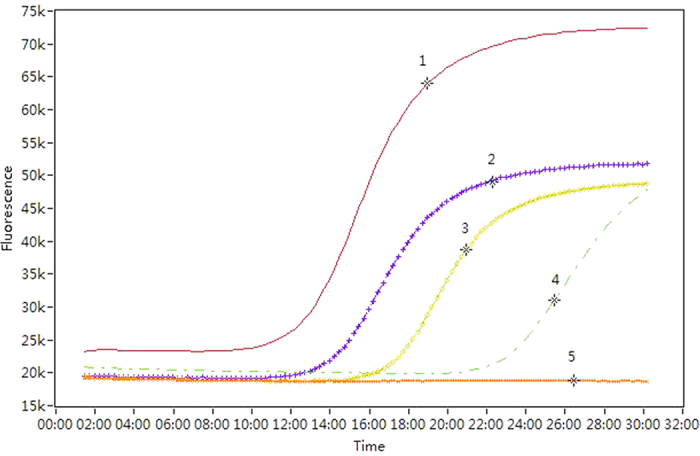
Example of loop-mediated isothermal amplification (LAMP) detection of phytoplasma showing positives in the heads of field-collected insects. (1) = positive control; (2) = *Zophiuma pupillata;* (3) = *Lophops saccharicida* (nymph); (4) = *Colgar* sp.; and (5) = negative control. Time axis uses minutes.

**Figure 2:**
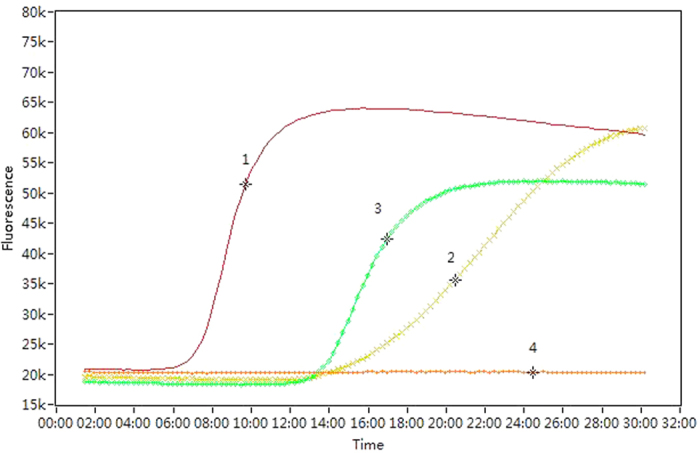
Example of loop-mediated isothermal amplification (LAMP) detection of phytoplasma showing positives in the sucrose media fed upon by field-collected insects. (1) = positive control; (2) = unidentified Zoraidini species; (3) = *Zophiuma pupillata*; and (4) = negative control. Time axis uses minutes.

Six of the 11 taxa tested: *Zophiuma pupillata*, *Lophops saccharicida*, the unidentified Zoraidini species, unidentified Ricaniidae species 1, *Taparella amata* and *Colgar* sp. were able to transmit phytoplasmas to the sucrose solution. Proportional incidence of positives from the solution which fed upon by positive insect samples did not differ significantly among taxa (*χ*^*2*^ = 4.266, *P* = 0.512) ranging from 0.333 for *L. saccharicida* to 0.100 for the *Colgar* sp. The adults and nymphs of *L. saccharicida* were tested separately and both life stages showed evidence of transmitting phytoplasmas to the feeding solution with an overall rate of positives of 0.333. Proportional incidence of positives from the adults and nymphs of *L. saccharicida* did not differ for head tissue samples (*χ*^*2*^ = 0.217, *P* = 0.642) or for feeding solution samples (Continuity Correction ^b^, *χ*^*2*^ = 0.938, *P* = 0.333). All taxa had a proportional incidence of phytoplasma detection in the head tissues of field-caught specimens of at least 0.077. Detection rate was greatest for the 58 individuals of *Z. pupillata* which yielded 31 positive tests. Two taxa, the unidentified Flatidae species 1 and unidentified Ricaniidae species 2 did not transmit phytoplasma to the feeding solution despite their head tissue testing positive for phytoplasma with a high incidence (>0.420) (Table 2). Proportional incidence of positives from the head tissue assays differed significantly among taxa (*χ^2^* = 64.890, *P* < 0.001) ranging from 0.667 for unidentified Ricaniidae species 2 to 0.077 for *Colgar* sp. Three additional unidentified species representing Derbidae and Cicadellidae consistently tested negatively for phytoplasma though only the derbid was represented by a sample size greater than single digits (Table 2). All of the taxa that assayed positive for the presence of BCS phytoplasma in the feeding medium also tested positive in head tissues. Representative positive results from the LAMP assay obtained from insect samples are shown in Fig. 1. This shows a rapid increase in fluorescence in positive samples from 10 to 20 min of the start of the reaction. The semi-quantitative nature of the assay is shown in several of the curves. In samples where the fluorescence increases earlier and more rapidly reflects a higher titre in the initial sample such as that shown for *Z. pupillata*. Representative positive results from the LAMP assay obtained from feeding medium samples are shown in Fig. 2.

### Nested PCR and sequencing

**Figure 3:**
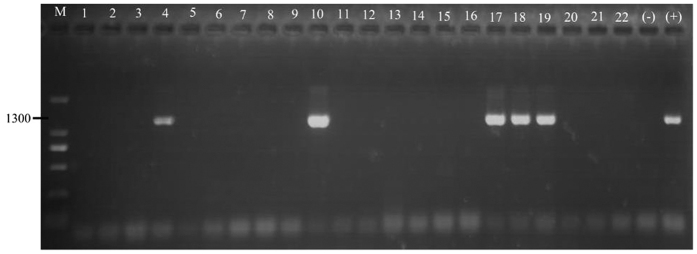
Example of nested PCR with primers R16F2n/R2 for the detection of phytoplasma in the heads of field-collected insects. Numbers 1–7 represent unidentified Zoraidini species; Numbers 8–16 represent *Colgar* sp.; (17) = *Zophiuma pupillata*; (18) = unidentified Ricaniidae species 2, Numbers 19–22 represent unidentified Ricaniidae species 1; M = DL 2000 marker; (+) = positive control; and (−) = negative control.

The unidentified Zoraidini species, *Colgar* sp*., Z. pupillata*, unidentified Ricaniidae species 1 and 2 tested positive for phytoplasma DNA presence in the head tissues by N-PCR assays (Fig. 3). These data are in accordance with the LAMP assays for the heads of these taxa.

**Figure 4:**
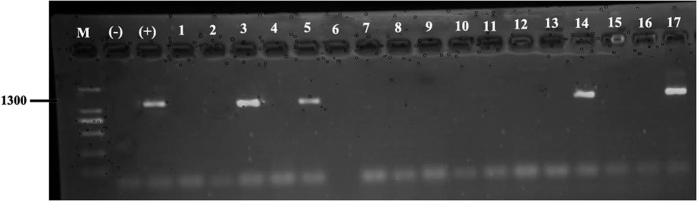
Example of nested PCR with primers R16F2n/R2 for the detection of phytoplasma in the sucrose media fed upon by field-collected insects. Numbers 1–3 represent unidentified Zoraidini species; Numbers 4–8 represent *Lophops saccharicida* (nymph); Numbers 9–14 represent *Colgar* sp.; Numbers 15–17 represent *Zophiuma pupillata;* M = DL 2000 marker; (+) = positive control; and (−) = negative control.

Sucrose solutions which were fed upon by the unidentified Zoraidini species, *L. saccharicida*, *Colgar* sp., and *Z. pupillata* also tested positive for phytoplasma DNA by N-PCR assays (Fig. 4).

**Figure 5:**
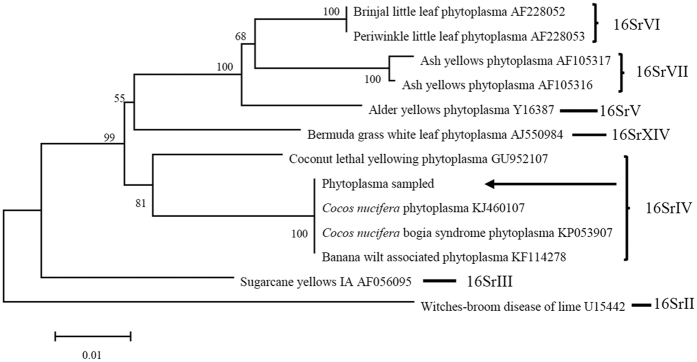
Phylogenetic distance tree of phytoplasmas constructed using the neighbor-joining method by comparing 16S rDNA sequences selected from GenBank and 16S rDNA sequence of sampled. Multiple alignments were performed with ClustalW and edited to a specific length of 1339 bp before constructing the distance tree. Numbers above the branches are confidence values obtained from 1000 bootstrap replicates. Sampled phytoplasma in PNG is shown by arrow as a member of 16SrIV group.

Sequencing results showed that the partial 16Sr DNA sequences matched 100% with ‘*Cocos nucifera*’ BCS phytoplasma. The homology matrix of sequences of phytoplasmas from different host plants supported BLAST comparisons. It showed that the phytoplasma isolated from insect heads and feeding medium samples in the present study have 100% homology and 100% coverage with ‘*Cocos nucifera*’ BCS phytoplasma sequences from GenBank (Fig. 5).

### The detection limit of loop-mediated isothermal amplification

The detection limit of LAMP was examined using DNA extracted from *Z. pupillata*. The initial DNA concentration was 30.2 ng/μl and the LAMP technique was capable of detecting phytoplasma in DNA after five 1:10 dilutions (10^−5^), equating to a concentration of 0.302 pg/μl in the original.

## Discussion

Surveys of insects found on and around affected plants and focusing on the phloem-feeding Hemiptera taxa are the first step towards determining vectors of a given phytoplasma disease. Transmission trials have been considered the only method of positively identifying the vectors in a phytoplasma pathosystem because detection of the pathogen in the insect is not a reliable indicator of its vector status. Vector transmission trials for coconut lethal yellowing have been carried out since the 1960s, however, very few have been conclusive^40^. There have been a great number of unsuccessful palm phytoplasma vector transmission trials^3^. To date the only positively identified vectors of LYD are the planthoppers *Haplaxius crudus* (van Duzee)^41^ (Cixiidae) previously named *Myndus crudus*, in Florida^42^ and the derbid *Proutista moesta* (Westwood) in India^43^. Of these two families, Cixiidae were absent from samples in the present study whilst Derbidae was represented by two unidentified species, neither being *P. moesta*

Our results show that six Hemiptera taxa and their feeding solutions were positive for the BCS phytoplasma. *Z. pupillata* showed a high proportion of individuals that tested positive in assays of head tissue and feeding medium samples. Combined with the fact that this species was one of the more commonly caught Hemiptera and present on all three BSC-affected sites it is relatively likely to be an important vector of the BCS phytoplasma. The other member of the Lophopidae present in samples, *L. saccharicida,* was also common, though not uniformly across sites. This too gave positive results in molecular assays. For this species, nymphs were collected from the foliage of BCS-affected species including banana and a significant proportion of these (as well as adults) were positive for phytoplasma DNA showing that both life stages are capable of acquiring the pathogen. Auchenorrhyncha are efficient vectors of phytoplasmas in part because they are hemimetabolous; thus, nymphs and adults feed similarly and are in the same physical location, often, both immature stages and adults can transmit phytoplasmas^17^. An example is the adults and nymphs of the brown marmorated stink bug, *Halyomorpha halys* Stål (=*H. mista* Uhler), can transmit witches’ broom phytoplasma to *Paulownia* spp. trees in Asia^44^. Though nymphs are wingless and this constrains their capacity to disperse phytoplasmas widely after acquisition, the vegetation structure of BCS infested sites, characterised by a polyculture of plant species with short plants such as banana and Areca and sago palms growing beneath taller coconuts allows the possibility of nymphs simply falling from tall plants to shorter ones beneath and effecting transmission without flight. The fact that an additional four species were shown to be capable of introducing phytoplasma DNA into their feeding medium suggests that BCS is potentially transmitted by an unusually wide range of vectors. No other vector testing for coconut lethal yellowing has identified more than one or two putative vector species. Some phytoplasmas are known to be transmitted by multiple insect species^17^,^45^,^46^. For example, chrysanthemum yellows (CY) phytoplasma is successfully transmitted by three leafhoppers: *Euscelidius variegatus, Macrosteles quadripunctulatus, and Euscelis incisus*^47^. In addition, Flavescence dorée phytoplasma (FDP) is transmitted by five leafhoppers: *Anoplotettix fuscovenosus*, *E. variegatus*, *E. incisus*, *O. albicinctus* and *A. laevis*^30^,^10^. This result has important practical implications for management of BCS. If transmission tests can be conducted, they can focus on the putative vector species identified in the present study. If those tests establish that there are multiple vectors, control approaches with a high degree of species specificity such as biological control and pheromone-based trapping (whether lure-and-kill or monitoring) would be challenging to implement in this situation. Alternatives such as host plant resistance to the pathogen and destruction of infected palms may be more appropriate but have clear disadvantages such as long development time and socio-economic impact, respectively.

Two other Hemiptera species in the present study (the unidentified Flatidae species 1 and the unidentified Ricaniidae species 2) had detectable, phytoplasma DNA in the head tissue suggesting that the pathogen had overcome the gut physical barrier but, because they were not detected in the feeding medium, are unlikely to be competent vectors. For the latter taxon, however, only three insects were tested and, though this low representation in field samples suggests a low risk of being an important vector, more exhaustive sampling and associated molecular testing would be necessary to rule it out as a potential vector. A similar need for wider testing applies to the additional three taxa that returned no positives in assays of feeding medium or head tissue before they can be discounted with confidence.

The most commonly reported vectors of phytoplasmas are members of the Hemiptera families: Cicadellidae, Derbidae, Flatidae, Cixiidae, Psyllidae, Cercopidae, Delphacidae and Meenoplidae^17^. Whilst the first three listed of these families were present in field samples from BSC affected sites, no positive DNA detections were made for the total of five Cicadellidae individuals. In addition to the positive DNA detections for members of the Derbidae and Flatidae, phytoplasma DNA was detected in specimens of additional families, the Lophopidae and Ricaniidae. Weintraub and Beanland listed 92 confirmed vector species which belonged to Derbidae, Flatidae, Cixiidae, Psyllidae, Pentatomidae, Tingidae, Cicadellidae and Delphacidae^17^.

The loop mediated isothermal amplification (LAMP) technique is becoming increasingly useful in the life sciences as it is a rapid diagnostic tool capable of processing large numbers of samples^48^,^49^,^50^,^51^. The method for determining which insects are likely to be vectors based on assaying for phytoplasma DNA in the sucrose solution upon which insects have fed, shows significant promise when used in tandem with LAMP assays of individual samples. In this study, LAMP detected phytoplasma DNA at a concentration of 0.302 pg/μl and other research has shown that LAMP is more sensitive than nested PCR^52^,^53^,^54^,^55^. The speed of the LAMP assay was also advantageous, where results were produced within a one hour when compared to the nested PCR technique, which can take 9 hours to complete. The LAMP method is a rapid diagnostic tool for glasshouse and laboratory studies and offers the potential to be applied in the field^56^. The 16S gene region used in this assay is highly conserved among phytoplasmas and so the design of primers using other gene regions or the use of whole genome sequencing would improve the discriminatory power of the assay. The findings of this study suggest that the use of LAMP for the detection of phytoplasma DNA is a valuable tool for the understanding and subsequent management of BCS. We anticipate the coupling of LAMP assays with feeding medium tests will prove useful in future phytoplasma studies as a way to determine which species need to be included in transmission tests, especially when large, perennial species such as palms are involved because these necessitate the use of large and expensive cages.

## Materials and Methods

### Collection and screening of insects

**Figure 6:**
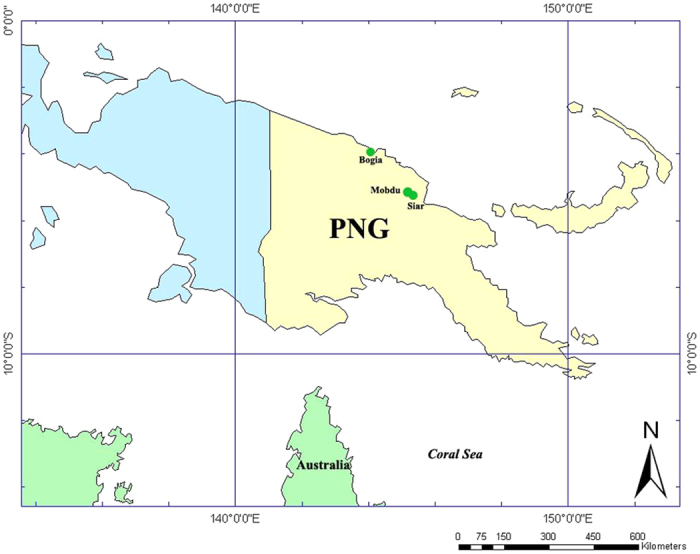
Map of the geographic locations of collection sites in Papua New Guinea. Drawn using ArcGIS10.0 (http://www.esri.com/software/arcgis/).

Live Hemiptera insects were collected in individual vials from crop and non-crop plants, with and without symptoms, three BCS-affected sites at Mobdu, Siar and Bogia (Fig. 6) in Madang Province in PNG (Table 1). The location map of sites (Fig. 6) was generated by ArcGIS 10.0 software (Esri, Redlands, California, USA; http://www.esri.com/software/arcgis/arcgis-for-desktop). Insects were held in an insulated (but non-chilled) box and returned promptly to the laboratory where they were transferred to individual white Eppendorf tubes (1.5 ml). To provide ventilation, the bottom ends of the tubes were previously removed and replaced with a cotton wool plug. The original caps of tubes were replaced with yellow-coloured caps to be more attractive to insects and encourage their feeding. The cavity within each cap was filled with 200 μl of 5% sucrose in ddH_2_O and covered with a Parafilm membrane that contained the liquid and separated it from the live insect. Tubes were kept at 25 °C for 48 to 72 h in a horizontal position with the yellow cap facing natural light to further promote insect attraction. Insects were then placed individually into 100% ethanol and the feeding solution sample placed individually into 200 μl of 100% ethanol.

### Extraction of DNA from insect specimens and feeding medium solution

DNA was extracted from insect heads using the Qiagen DNeasy Blood & Tissue Kit and from the sucrose solution according to Zhang *et al*. (1998). The sucrose solution was centrifuged at 12,000 × g for 20 min. Ten microliters of 0.5 N NaOH was added to the pellet followed by 20 μl of 1 M Tris-HCl buffer, pH 8.0, containing 1% sodium dodecyl sulfate and 20 mM EDTA. The mixture was incubated at 65 °C for 20 min, then precipitated with two volumes of ethanol, and kept at −20 °C for at least 30 min. DNA was precipitated by centrifugation at 12,000 × g for 20 min and resuspended in 20 μl of ddH_2_O.

### Design of LAMP primers

Primers for the LAMP assay were designed based on the partial sequence of the 16S ribosomal RNA gene region of *C. nucifera* tissue testing positive for BCS (GenBank sequence KP053907.1, 1808 bp). The positive control was DNA extracted from diseased coconut tree tissue which was collected in Dugumar Village, Bogia District, Papua New Guinea in June 2008 57. LAMP Designer 1.10 (Premier Biosoft International) was used to design the 6 primers consisting of the forward outer (F3), backward outer (B3), forward inner (FIP), backward inner (BIP) and the forward and reverse loop (Loop F and Loop B, respectively). Primers were synthesized by GeneWorks Pty Ltd, Australia and are detailed in Table 3.

### LAMP and nested-PCR detection of phytoplasma DNA in insects and feeding medium

The LAMP reaction was performed in a final volume of 12.5 μl consisting of 5 pM each of the F3 and B3 primers, 20 pM each of FIP and BIP primers, 10 pM each of LoopF1 and LoopB1 primers, 7.5 μl of 1x Isothermal Master Mix (OptiGene Ltd.) and 2.5 μl of sample template. All reactions were performed using a Genie^®^ II (OptiGene Ltd.) at 65 °C for 30 min, followed by annealing at 98–80 °C (ramping at 0.05 °C^−s^). For each run, a reaction containing a PCR product (amplified with a nested-PCR as described below) positive for BCS phytoplasma served as the positive control (as described above). The PCR product was diluted 1:100 before use. A reaction with no template DNA served as a negative control. A chi-square test was used to compare the proportional rate of phytoplasma DNA detections among taxa (excluding taxa with zero positives and using *L. saccharicida* species total number) for the head tissue assays and the feeding medium of positive insects assays. A separate chi-square test was used to compare the proportional rate of phytoplasma DNA detections between adult and nymph of *L. saccharicida* for the head tissue assays and the feeding medium of positive insects assays. For the latter, the small sample size required the use of a continuity correction.

Representative samples that tested positive for phytoplasma DNA in the LAMP assay were also analysed with N-PCR amplification with the first pair of primers R16mF2/R1 and the second pair of primers R16F2n/R2 (Table 3) using a BIO-RAD T100^TM^ Thermal Cycler. In the N-PCR assay, PCR products initially amplified using the universal primers pair R16mF2/R1 were diluted to 1/20 with sterile deionized water and used as template for the N-PCR amplification using the second primers pair R16F2n/R2^58^. The reaction was performed in a final volume of 25 μl consisting of 1 μl of 10 μM each primer, 12.5 μl 2x GoTaq^®^ Green Master Mix (Promega USA) and 1 μl of sample template. The reaction mixture was adjusted to 25 μl using sterile distilled water. Thirty-five PCR cycles were conducted with the following parameters: 1 min (2 min for the first cycle) denaturation at 94 °C, annealing for 2 min at 60 °C (55 °C for the reactions using N-PCR condition), and extension for 3 min (10 min for the final cycle) at 72 °C. Double distilled water was used as negative control and the previously described positive control. Amplicons were analyzed by electrophoresis using a 1% agarose gel (DYY-100 electrophoresis apparatus), stained with GoldView and visualized under UV light (BioSpectrum^®^ MultiSpectral Imaging System).

### Phylogenetic analysis

N-PCR positive products were sent to Biotech Corp (Biosune Co. Ltd., Shanghai, China) for purifying and sequencing. These positive samples were from 6 insect species including *Z. pupillata*, *L. saccharicida* (nymph), unidentified Zoraidini species, *Colgar* sp., unidentified Ricaniidae species 1 and 2 as well as the sucrose medium which was fed upon by *L. saccharicida* (nymph), the unidentified Zoraidini species and *Colgar* sp. Multiple sequences were aligned and edited to a final, consistent length of 1333 bp using program DNAMAN and analyzed with BLAST at the National Centre for Biotechnology Information (NCBI).

A phylogenetic distance tree was constructed by the neighbor-joining method comparing 16S rDNA sequence of sample with those of 16S/23S spacer sequences from 12 diverse phytoplasmas from GenBank using MEGA5.1 with 1000 bootstrap replicates^59^(Fig. 5). Multiple sequence alignments were performed with Clustal W and edited to a consistent length of 1339 bp before constructing the distance tree^60^.

### Determining the detection limit of loop-mediated isothermal amplification (LAMP)

A DNA sample of the positive control was quantified using a NanoDrop2000 spectrophotometer. The DNA was diluted 1:10, seven consecutive times, and tested for phytoplasma presence by LAMP as described previously. Double distilled water was used as negative control.

## Additional Information

**How to cite this article**: Lu, H. *et al*. Determining putative vectors of the Bogia Coconut Syndrome phytoplasma using loop-mediated isothermal amplification of single-insect feeding media. *Sci. Rep.* **6**, 35801; doi: 10.1038/srep35801 (2016).

**Publisher’s note:** Springer Nature remains neutral with regard to jurisdictional claims in published maps and institutional affiliations.

## Figures and Tables

**Table 1:** Numbers of Hemiptera taxa collected from BCS-affected sites in Madang Province in PNG.

Taxon	Site			Total
	MOBDU	SIAR	BOGIA	
**Lophopidae**				
*Zophiuma pupillata*	17	5	36	58
*Lophops saccharicida* (adult)	38	2	0	40
*Lophops saccharicida* (nymph)	18	5	2	25
**Derbidae**				
Unidentified Zoraidini species	70	24	40	134
Unidentified Derbidae: Derbinae: Cenchreini	20	0	0	20
**Ricaniidae**				
Unidentified Ricaniidae species 1	46	5	4	55
Unidentified Ricaniidae species 2	1	2	0	3
**Flatidae**				
*Taparella amata* (Walker)	21	86	0	107
*Colgar* sp.	73	46	11	130
Unidentified Flatidae species 1	0	0	50	50
**Cicadellidae**				
Unidentified Cicadellidae species 1	4	0	0	4
*Paramesodes* sp.	0	0	1	1

**Table 2:** List of insect taxa ranked for likelihood as BCS vectors based on proportional rate of phytoplasma DNA in feeding media and insect head tissues.

Insect taxon	Number tested	Proportion of positive in insects (n, no. of positive, in brackets)	Proportion of positive in the feeding medium of positive insects (n, no. of positive, in brackets)
*Lophops saccharicida* (adult + nymph total)	65	0.231 (15)	0.333 (5)
*Lophops saccharicida (*nymph)	25	0.200 (5)	0.600 (3)
*Lophops saccharicida* (adult)	40	0.250 (10)	0.200 (2)
*Zophiuma pupillata*	58	0.534 (31)	0.290 (9)
Unidentified Ricaniidae species 1	55	0.145 (8)	0.250 (2)
*Taparella amata*	107	0.187 (20)	0.200 (4)
Unidentified Zoraidini species	134	0.224 (30)	0.133 (4)
*Colgar* sp.	130	0.077 (10)	0.100 (1)
Unidentified Flatidae species 1	50	0.420 (21)	0 (0)
Unidentified Ricaniidae species 2	3	0.667 (2)	0 (0)
Unidentified Derbidae: Derbinae: Cenchreini	20	0 (0)	—
Unidentified Cicadellidae species 1	4	0 (0)	—
*Paramesodes* sp.	1	0 (0)	—
Chi square (for *n,* excluding taxa with zero positives)		64.890	4.266
*P* (for *n,* excluding taxa with zero positives)		<0.001	0.512

**Table 3:** Oligonucleotide primers for the detection of phytoplasma DNA by LAMP and by nested PCR.

Primer	Sequence (5′-3′)	Length	Tm(°C)
F3	CGCCACATTAGTTAGTTGGTA	21	60.1
B3	TTCATCGAATAGCGTCAAGG	20	60.1
FIP	GTTTGGGCCGTGTCTCAGTGCCTACCAAGACGATGATG	38	
BIP	TACGGGAGGCAGCAGTAGGAGTACTTCATCGTTCACGC	38	
LoopF	GTGGCTGTTCAACCTCTCA	19	62.0
LoopB	AACTCTGACCGAGCAACG	18	62.1
R16mF2	CATGCAAGTCGAACGGA	17	60.0
R16mR1	CTTAACCCCAATCATCGAC	19	60.0
R16F2n	GAAACGACTGCTAAGACTGG	20	55.0
R16R2	TGACGGGCGGTGTGTACAAACCCCG	25	55.0
